# Protocol of an Efficacy Study on Nursing Consultation in Weight Management and Metabolic Syndrome Based on the Carbohydrate–Insulin Theory in Individuals with First-Episode Psychosis

**DOI:** 10.3390/nursrep15010003

**Published:** 2024-12-27

**Authors:** Lander Madaria, Claudia Aymerich, Ana Catalan, Miguel Angel Gonzalez-Torres

**Affiliations:** 1 Psychiatry Department, Basurto University Hospital, 48013 Bilbao, Spain; claudia.aymerichnicolas@osakidetza.eus (C.A.); ana.catalanalcantara@osakidetza.eus (A.C.); miguelangel.gonzaleztorres@osakidetza.eus (M.A.G.-T.); 2 Biocruces Bizkaia Health Research Institute, 48903 Barakaldo, Spain; 3 Neuroscience Department, University of the Basque Country, UPV/EHU, 48940 Leioa, Spain; 4 Centro de Investigación en Red de Salud Mental (CIBERSAM), 28029 Madrid, Spain; 5 Department of Psychosis Studies, Institute of Psychiatry, Psychology and Neuroscience, King’s College London, London SE5 8AB, UK

**Keywords:** nursing, mental health, metabolic syndrome, insulin resistance, psychosis

## Abstract

**Background**: Individuals with severe mental illness live, on average, up to 30 years less than the general population, with cardiovascular disease being the leading cause of death. Metabolic syndrome (MetS) plays a significant role in this, making it crucial to manage this issue in individuals with psychosis at the onset of the illness. The approach to managing this issue has evolved from a focus on calorie counting to a deeper understanding of hormone function, particularly the role of insulin resistance in MetS. Therefore, incorporating this perspective into mental health nursing consultations with individuals experiencing psychosis is of great interest. **Methods**: In accordance with the SPIRIT guidelines, an open randomized clinical trial is proposed, involving patients from a first-episode psychosis program. **Results**: The primary outcome will be significant weight loss (≥5%). Secondary outcomes will include changes in metabolic parameters, psychopathological status, quality of life, and physical activity. Participants will be assigned to two groups: one group will attend a series of six previously manualized nursing consultations, while the other will continue with their usual treatment. Results will be evaluated at six months and one year. **Conclusions**: This study will determine whether a mental health nursing consultation based on the carbohydrate–insulin model of obesity is effective in reducing weight and the risk of MetS in individuals with early-onset psychosis. This study was retrospectively registered on Clinical Trials—NCT06650943.

## 1. Introduction

The life expectancy of individuals with severe mental disorders is estimated to be 20 [1] to 30 [2] years shorter than that of the general population, with evidence suggesting that this disparity has widened in recent decades [3].

Cardiovascular and metabolic diseases are the main causes of death in this population group [4], and having a diagnosis of psychosis is considered a risk factor for the development of these diseases [5]. It is also a risk factor for death from cardiovascular or cerebrovascular events [6].

Metabolic syndrome (MetS), a common cause of cardiovascular and metabolic diseases, develops in one out of every three individuals experiencing psychosis, nearly doubling the odds compared to the general population [7,8]. MetS includes central obesity, increased fasting glucose, dyslipidemia, and hypertension, with insulin resistance playing a central role in various tissues of the body [9,10]. Different definitions have been proposed with similar criteria to diagnose MetS, which involve waist circumference, blood triglyceride levels, HDL cholesterol, fasting glucose, and systolic and diastolic blood pressure biomarkers [11,12,13].

In people who experience psychosis, one of the causes of MetS is the deregulation of glucose metabolism induced by antipsychotic treatment, especially second-generation antipsychotics [14]. In people who have experienced a first psychotic episode, the first year of treatment is crucial for weight gain and the establishment of metabolic alterations [15], with early insulin resistance being a predictor of weight gain and increased waist circumference [16], related to the alteration of hunger regulation [17]. With some exceptions such as Ziprasidone and Lurasidone, all antipsychotics cause significant weight variation, with a greater effect seen in the case of Clozapine and Olanzapine [18].

There is also evidence suggesting that the altered metabolic parameters may not only be due to antipsychotic treatment [19], but also involve other systems beyond the central nervous system in individuals with psychosis [20]. Poor dietary habits [21] or sedentary behavior, closely related to the negative symptoms of psychotic processes such as a lack of motivation or cognitive deterioration, have also been associated with MetS. Additionally, low economic income has been correlated with worse metabolic parameters [22].

For this reason, reference organizations such as the National Institute for Health and Care Excellence (NICE), in its guidelines for adult [23] and child [24] psychosis, and the International Physical Health in Youth (iphYs) working group [25], recommend monitoring these aspects in people who experience early-onset psychosis and implementing approaches to modify lifestyle habits in nutrition and physical activity.

The approach to metabolic disorders such as obesity and type 2 diabetes mellitus has been evolving in recent years [26]. The shift has been from a calorimetric perspective focused on calorie counting to an approach that emphasizes modifying the hormonal response triggered by various foods [27]. The carbohydrate–insulin model posits that the glycemic impact of nutrition triggers a hormonal response, particularly involving insulin, which in the long term affects weight variability and the development of metabolic and cardiovascular pathologies. Glucose spikes contribute to elevated insulin levels in the blood, hindering the use of fat as an energy source and promoting weight gain [28]. It has been observed that decreases in fasting insulin levels precede weight loss [29]. Moreover, the glycemic response to different meals could influence appetite and energy intake [30].

Given the evolving understanding of metabolic disorders, the role of healthcare professionals, including mental health nurses, needs to adapt to these changes. Mental health nurses, who serve as key health educators in individuals with mental health problems [31], have traditionally focused on providing nutritional education aimed at caloric balance, encouraging patients with psychosis to consume fewer calories and increase physical activity [32], or on identifying metabolic syndrome [33].

In studies employing a multidisciplinary approach, interventions that include an individualized follow-up [34] and a psychotherapeutic component have shown better results in managing MetS compared to purely educational group formats [35].

However, there is no evidence regarding whether nursing interventions that use a hormonal reference model are effective for weight management and reducing the risk of metabolic syndrome in individuals with recent onset psychosis.

Therefore, the aim of this study is to evaluate the effect of a nursing intervention based on the carbohydrate–insulin model on weight loss and the management of MetS risk in people with recent onset psychosis. Secondary aims include evaluating the effect of the intervention on (a) metabolic parameters related to MetS, (b) psychopathological state, (c) physical activity level, and (d) quality of life.

## 2. Methods

### 2.1. Design

This protocol is designed as an open-label, randomized clinical trial involving a group of patients in a first-episode psychosis (FEP) program, who will attend a series of nursing consultations focused on healthy habits aimed at addressing metabolic syndrome, in addition to their usual follow-up. The control group will consist of patients from the same program who will attend only routine nursing evaluation consultations.

The first author (L.M.) will conduct the nursing consultations, based on a previously developed guide that structures and serves as a reference framework for the intervention.

This study was retrospectively registered on Clinical Trials—NCT06650943.

### 2.2. Setting

This intervention will take place within a hospital-based Early Intervention Program (EIP) for FEP patients at Basurto University Hospital (HUB) in Bilbao, Spain.

This EIP is a public program serving a total population of 347,000 people, with an annual attendance of 150 patients. Patients enter this program either through admission to the short-stay inpatient unit following a FEP, or by being treated in the Emergency Service for the same reason.

### 2.3. Participants

The inclusion criteria for the present study will include the following: (a) patients admitted to the EIP due to a (b) FEP within the last 5 years, (c) with a diagnosis of psychosis from the F2 spectrum or an affective disorder with psychotic symptoms as per the ICD-10 [36].

Exclusion criteria will include (a) cognitive inability to learn or a comorbid diagnosis of an intellectual disability that interferes with the study procedures, (b) a comorbid diagnosis of neurological pathology, (c) the presence of language or comprehension alterations that prevent accurate data collection, (d) being prescribed a hypoglycemic drug before or during the study, or (e) refusal to participate. No patients will be excluded based on their special dietary needs (e.g., celiac disease, veganism, special diets) or a diagnosis of MetS before the start of the intervention. The primary consideration will be the individual’s desire to improve their weight or metabolic status. A participant will be considered a drop-out if they miss 2 or more of the first 6 consultations, are admitted to the hospital due to psychotic relapse, and/or experience psychopathological decompensation characterized by a score of 5 or higher on items P1 (delusions), P2 (conceptual disorganization), P3 (hallucinatory behavior), P6 (suspiciousness, persecution), P7 (hostility), and/or G8 (uncooperativeness) on the Positive and Negative Symptoms Evaluation Scale (PANSS) [37], and/or exhibit clinically significant self-directed or outwardly aggressive behavior.

### 2.4. Randomization and Treatment Allocation

Subjects will be selected during their psychiatry consultations following the stabilization of their pathology. If they meet the inclusion/exclusion criteria, they will be informed about the possibility of participating in the study, including its content and purpose. After being provided with an informed consent form and signing it, they will be randomly assigned to either the experimental or control group. Randomization will be conducted using a computer-generated random list. As participants are offered the opportunity to join the study, they will be assigned a number from the list in sequential order. The psychiatrist who refers the participants will not have access to the randomization list, ensuring allocation concealment. On the other hand, the psychiatrist responsible for administering the psychometric scales will be blinded to the intervention.

### 2.5. Intervention

The study intervention will consist of a series of 8 nursing consultations with a specialized mental health nurse, who is trained in teaching healthy habits to individuals with psychosis and understands the unique factors influencing this group. The nurse will also be skilled in using the therapeutic relationship as a tool for habit modification.

During the first two months, 4 consultations will be held, focusing on educating patients about dietary habits based on the carbohydrate–insulin theory of obesity and physical activity, following a manualized intervention previously designed by a team composed of a mental health nurse, a nutrition and dietetics graduate, and a psychiatrist. This guide has been designed based on the consensus report of the American Diabetes Association for nutrition therapy for diabetes and prediabetes [26], as well as incorporating insights from clinical experience with individuals with a first episode of psychosis. The next two visits will take place 3 and 4 months after the first visit, respectively. Visit 7 will be held after 6 months to evaluate the intervention’s effectiveness, and visit 8 at 12 months. During all visits, weight will be monitored as a reinforcement element.

At the beginning of the study, and at 6 and 12 months from the start of the intervention, a battery of tests will be conducted, including analytical samples, vital signs, anthropometric measurements, and scales to assess psychopathology, quality of life, and level of physical activity.

As shown in Table 1, each consultation will have a central topic. The first session will cover the objectives of the intervention, emphasizing the achievement of greater well-being and the prevention of various cardiometabolic illnesses. The second session will explain the importance of food quality over calorie counting. In the third session, patients will be educated on identifying different macronutrients, their functions, and the relationship between insulin and weight regulation. The fourth session will provide various tips and types of diets to help patients incorporate this model into their own situations. The fifth session will focus on physical activity, highlighting the most efficient types for fat loss and the improvements that patients can implement. The sixth consultation will involve a follow-up to assess any difficulties in making changes and refine strategies to overcome them. After this consultation, the intervention will be concluded. The overall approach will focus on the progressive and flexible acquisition of habits, aiming to integrate changes into the patient’s daily life. This will occur within the therapeutic relationship established between the mental health nurse and the patient, utilizing a psychoeducational and non-blaming approach.

The consultations will use a previously developed guide, which will be provided to patients to allow them to review the information discussed during the sessions.

To improve adherence to the intervention protocol, participants will be called by phone two business days before their visits.

### 2.6. Control Group

Both the intervention group and the control group will continue their usual follow-ups within the EIP, attending consultations with their reference psychiatrist and receiving pharmacological or psychotherapeutic treatment as they did before the intervention. The control group will not receive any specific nursing consultations (beyond the administration of long-acting injectable antipsychotics where appropriate) or dietary or lifestyle advice during the follow-up. If patients in the control group require treatment or assistance for metabolic problems, they will be referred to primary care or endocrinology professionals, as was done previous to the intervention implementation.

### 2.7. Instruments and Variables

Sociobiographic data will be collected, including sex, age, marital status, living arrangement, educational level, medical diagnosis, time since the beginning of treatment, substance use, antipsychotic type and dose, mood stabilizer use, and whether patients are assisted by a primary caregiver (considered as such if they assist in 4 of the first 6 consultations).

The primary outcome will be weight loss, with a reduction of 5% considered clinically significant [38]. Secondary outcomes will include the establishment or non-establishment of metabolic syndrome according to the International Diabetes Federation (IDF) criteria [12], which consist of abdominal obesity (abdominal circumference ≥ 94 cm in men and ≥80 cm in women) plus two of the following four factors: (i) elevated blood triglycerides (≥150 mg/dL or treatment for it); (ii) decreased HDL-c (<40 mg/dL in men and <50 mg/dL in women); (iii) high blood pressure (systolic blood pressure ≥ 130 mmHg and diastolic blood pressure ≥ 85 mmHg or antihypertensive treatment); and (iv) elevated fasting glucose (≥100 mg/dL or previous diagnosis of type 2 diabetes).

Anthropometric variables (weight, height, BMI, waist circumference) and vital signs (resting blood pressure) will be measured using calibrated equipment provided by the hospital.

Laboratory values (fasting blood glucose, glycosylated hemoglobin, triglycerides, HDL, HDL/LDL ratio, C-reactive protein) will be collected in accordance with hospital policy.

To measure psychopathology, the Positive and Negative Syndrome Scale (PANSS) will be used. This scale consists of 30 items divided into three subscales: positive, negative, and general. The positive subscale measures symptoms such as hallucinations and delusions, the negative subscale measures symptoms such as flat affect and lack of motivation, and the general subscale measures symptoms such as agitation and hostility [37].

Depressive symptoms will be assessed using the Hamilton Depression Rating Scale (HAM-D), which consists of 17 items, with higher scores indicating greater severity of symptoms [39].

The EuroQol-5D (EQ-5D) scale will be used to evaluate a person’s subjective well-being in relation to their health. The scale consists of five dimensions: mobility, self-care, usual activities, pain/discomfort, and anxiety/depression [40].

Finally, the International Physical Activity Questionnaire (IPAQ) will be used to measure the level of reported physical activity. It consists of 8 questions that evaluate the amount and intensity of physical activity in different settings such as work, home, transportation, and leisure time. It also asks about vigorous and moderate physical activity, as well as sedentary activity [41].

### 2.8. Statistical Analysis

Categorical variables will be described as frequencies and percentages, while quantitative variables will be described as means and standard deviation (or medians and interquartile ranges where appropriate). Categorical variables will be compared using chi-square analysis or Fisher’s exact test, and quantitative variables will be compared using Student’s *t*-test, ANOVA, or the Mann–Whitney rank-sum test.

To compare changes in outcome variables between groups and over time, a multivariate mixed-effects model with repeated measures (linear or logit, depending on the characteristics of the variable) will be applied. Statistical significance will be assumed if the two-sided *p*-value is <0.05. The main analysis will assess the association between the study group and significant weight loss, defined as losing 5% of the original body weight.

To account for the potential effect of antipsychotics on weight and metabolic parameters, antipsychotics will be categorized as weight-increasing, weight-sparing, or as having non-conclusive effects. Sensitivity analyses will be conducted to examine the effect of each category on the outcomes. Categorization will be based on a review of previous evidence [42]. The use of weight-increasing antipsychotics in combination with weight-sparing antipsychotic use will be coded as weight-increasing antipsychotic use. The cumulative exposure of chlorpromazine-equivalent doses will then be calculated for each antipsychotic category in every patient [43].

Sensitivity analyses will also be conducted for each of the variables included in the previous section.

### 2.9. Power and Sample Size

We aim to detect a 5% difference in total weight, considering this to be clinically significant [36]. Using a power of 80%, a bilateral alpha of 5%, and assuming a standard deviation of 7.5% in an area where evidence is limited (weight loss in people who have experienced a first psychotic episode). A sample size of 74 participants will be required to detect this difference. Assuming a dropout rate of 20%, we aim to recruit a total of 88 participants, with 44 in each group.

### 2.10. Safety Assessments

Although no adverse effects are anticipated from the intervention, if they do occur, they will be recorded to the patients’ medical records and evaluated. In the event that risks are observed for participants or others, participation in the study will be suspended in those specific cases.

### 2.11. Data Collection and Management

A notebook will be created to record the data obtained in an orderly manner for each subject over time, and this data will subsequently be transferred to a unified database designed for this purpose. The samples and associated data will be coded. Therefore, the handling, communication, and transfer of such data will be governed by Organic Law 3/2018 of December 5th, on the protection of personal data and the guarantee of digital rights. Participants may access, correct, or cancel their data at any time. Only the research team and the health authorities, who are duty-bound to maintain confidentiality, will have access to all the data collected by the study, which will be stored securely and guarded by the principal investigator (PI). This study will be conducted in accordance with the Declaration of Helsinki and the rules of Good Clinical Practice. Participants and/or guardians will also be informed that this intervention is not part of the standard service offering and that any subject may withdraw from the study at any time upon request.

## 3. Conclusions

The results obtained from this study will provide valuable information on the changes in weight and key MetS parameters that occur following a nursing consultation designed to prevent and manage this condition in individuals with early-onset psychosis. If the results of this study are positive, the study should be replicated with a larger sample size and with different personnel implementing the intervention.

## Figures and Tables

**Table 1:** Schedule of enrolment, interventions and assessments.

	Enrolment	Allocation	Post-Allocation							
TIMEPOINT	0	0	w 1	w 3	w 5	w 8	w 12	w 16	w 24	w 48
ENROLMENT:										
Eligibility screen	X									
Informed consent	X									
Allocation		X								
INTERVENTIONS:										
Mental health nurse consultation			X	X	X	X	X	X		
Aims and giving patients handbook			X							
Calorie counting errors and food to avoid				X						
Insulin and fats, proteins and CH					X					
Tips and feeding protocols						X				
Physical activity type							X			
Follow-up and counselling								X		
Treatment as usual										
ASSESSMENTS:										
Weight	X	X	X	X	X	X	X	X	X	X
Anthropometric variables (height, blood pressure, lab test…)			X						X	X
Scales (PANSS, HAM-D, EQ-5, IPAQ)			X						X	X

## Data Availability

No data are available as this manuscript presents a study protocol.

## Public Involvement Statement

There is no public involvement in any aspect of this research.

## Guidelines and Standards Statement

This protocol is designed according to the Standard Protocol Items: Recommendations for Interventional Trials (SPIRIT) guidelines.
